# Semaphorin 5A drives melanoma progression: role of Bcl-2, miR-204 and c-Myb

**DOI:** 10.1186/s13046-018-0933-x

**Published:** 2018-11-19

**Authors:** Simona D’Aguanno, Elisabetta Valentini, Maria Grazia Tupone, Marianna Desideri, Marta Di Martile, Manuela Spagnuolo, Simonetta Buglioni, Cristiana Ercolani, Italia Falcone, Marco De Dominici, Michele Milella, Maria Giulia Rizzo, Bruno Calabretta, Carlo Cota, Andrea Anichini, Daniela Trisciuoglio, Donatella Del Bufalo

**Affiliations:** 1 Preclinical Models and New Therapeutic Agents Unit, IRCCS Regina Elena National Cancer Institute, Rome, Italy; 2 Oncogenomic and Epigenetic Unit, IRCCS Regina Elena National Cancer Institute, Rome, Italy; 3 Pathology Unit, IRCCS Regina Elena National Cancer Institute, Rome, Italy; 4 Medical Oncology 1, IRCCS Regina Elena National Cancer Institute, Rome, Italy; 5 Department of Cancer Biology, Sidney Kimmel Cancer Center, Thomas Jefferson University, Philadelphia, PA USA; 6 Dermatopathology Unit, IRCCS San Gallicano Dermatological Institute, Rome, Italy; 7 Fondazione IRCCS Istituto Nazionale dei Tumori, Milan, Italy; 8 Institute of Molecular Biology and Pathology, National Research Council, Rome, Italy

**Keywords:** Melanoma, Semaphorin 5A, Bcl-2, c-Myb, miR-204

## Abstract

**Background:**

Melanoma, the most aggressive form of skin cancer, is characterized by high rates of metastasis, drug resistance and mortality. Here we investigated the role of Semaphorin 5A (Sema5A) on the properties associated with melanoma progression and the factors involved in Sema5A regulation.

**Methods:**

Western blotting, qRT-PCR, Chromatin immunoprecipitation (ChIP) assay, immunohistochemistry of melanoma patient specimens and xenograft tissues, in vitro Transwell assay for cell migration and invasion evaluation, in vitro capillary-like structure formation analysis.

**Results:**

A significant correlation of Sema5A mRNA expression and melanoma progression was observed by analyzing GEO profile dataset. Endogenous Sema5A protein was detected in 95% of human melanoma cell lines tested, in 70% of metastatic specimens from patients affected by melanoma, and 16% of in situ melanoma specimens showed a focal positivity. We demonstrated that Sema5A regulates in vitro cell migration and invasion and the formation of vasculogenic structures. We also found an increase of Sema5A at both mRNA and protein level after forced expression of Bcl-2. By use of transcriptional and proteasome inhibitors, we showed that Bcl-2 increases the stability of Sema5A mRNA and protein. Moreover, by ChIP we demonstrated that Sema5A expression is under the control of the transcription factor c-Myb and that c-Myb recruitment on Sema5A promoter is increased after Bcl-2 overexpression. Finally, a concomitant decrease in the expression of Sema5A, Bcl-2 and c-Myb proteins was observed in melanoma cells after miR-204 overexpression.

**Conclusion:**

Overall our data provide evidences supporting the role of Sema5A in melanoma progression and the involvement of Bcl-2, miR-204 and c-Myb in regulating its expression.

**Electronic supplementary material:**

The online version of this article (10.1186/s13046-018-0933-x) contains supplementary material, which is available to authorized users.

## Introduction

Semaphorins (SEMAs), a large family of phylogenetically conserved proteins classified into eight classes, are involved in different physiological and developmental functions, including regulation of the nervous and immune systems and angiogenesis [1]. In the past years, several studies have shown that SEMAs and their receptors also regulate tumour growth and metastasis [2], but the mechanism of action in cancer, and particularly in melanoma, is not completely understood. Sema3A, Sema3B and Sema3F have been long considered inhibitors of tumor growth and metastasis [3–5], even if it has been reported that Sema3E inhibits tumor growth but promotes metastasis [6]. On the contrary, Sema6A positively correlates with melanoma migration [7] and Sema7A is associated with melanoma metastasis [8]. Controversial data exist regarding the role of Sema5A in cancer [9]. Reduced expression of Sema5A was observed in clinical samples of high-grade astrocytomas compared to normal brain tissue, and Sema5A was found to inhibit migration and invasion in human glioma cells by interacting with the plexin-B3 receptor and disrupting Rac1 activity [10]. Downregulation of Sema5A in tumor tissues was also reported in non-smoking women with non-small cell lung carcinoma and was associated with poor survival [11]. On the contrary, Sema5A was reported to promote invasion/metastasis in gastric cancer through activation of metalloprotease-9 (MMP-9) and urokinase-type plasminogen activator, and to increase pancreatic cancer metastasis via the Met receptor tyrosine kinase or the MEK/ERK pathway [11–15]. The ability of Sema5A to enhance metastases formation in some cancer models has been ascribed to its effect on angiogenesis: in vivo Matrigel plug assays demonstrated that Sema5A promotes angiogenesis and treatment of endothelial cells with recombinant extracellular domain of Sema5A enhanced endothelial cells proliferation, through Akt pathway activation, and increased migration through Met tyrosine kinase receptor and upregulation of MMP-9 [14].

In this study, we investigated the role of Sema5A in melanoma progression, and the molecular mechanism regulating its expression. We also focused on the functional relation between Sema5A and Bcl-2, an anti-apoptotic protein associated with melanoma progression, resistance to apoptosis and poor prognosis [16]. We previously demonstrated that, in addition to its canonical anti-apoptotic role, Bcl-2 is involved in multiple non-canonical functions, including melanoma metastasis, angiogenesis, and autophagy [17]. In particular, Bcl-2 overexpression in human melanoma cells increases in vitro and in vivo tumor progression-associated properties and angiogenesis [17–21] and promotes a cancer stem cell phenotype [22]. Moreover, treatment of melanoma cells with Bcl-2 antisense oligonucleotides induces antiangiogenic activity [23] and increases sensitivity to antineoplastic treatments [24]. We have also shown that Bcl-2 regulates the activity of transcription factors, such as microphthalmia-associated transcription factor, a master regulator of melanocyte and melanoma biology, and consequently its specific target genes, TRPM1, MLANA and miR-211 [25].

In this paper, we evidence Sema5A expression in metastatic specimens from melanoma patients, and we show that Sema5A expression modulates in vitro melanoma cell migration and invasion, activates the Akt/ERK pathway and is regulated by Bcl-2 and the miR-204/c-Myb axis.

## Methods

### Patients and tissue samples

Thirteen cases of metastatic melanoma specimens used to analyse Sema5A expression were obtained from melanoma patients surgically treated at the IFO-Regina Elena National Cancer Institute. The 13 metastatic specimens were obtained from the biopsies with different localization: 6 inguinal lymph nodes, 2 laterocervical lymph nodes, 1 parathyroid lymph node, 1 subscapularis lymph node, 2 brain and 1 lung metastatic lesions. We also analyzed Sema5A expression in specimens from in situ melanoma lesions (early melanoma lesions devoid the basement membrane invasion) from 12 patients collected at the IFO-San Gallicano Dermatological Institute. Three-micrometer sections of formalin-fixed paraffin-embedded tumor samples were cut on SuperFrost Plus slides (Menzel-Gläser, Braunschweig, Germany). Immunoreactions were revealed by Bond Polymer Refine Detection in an automated autostainer (Bond III, Leica Biosystems, Wetzlar, Germany) using Sema5A polyclonal antibody (#PA5–30884, ThermoFisher, Waltham, MA, USA). Diaminobenzidine was used as chromogenic substrate. The immunohistochemistry (IHC) results for Sema5A were recorded as positive when > 10% of the neoplastic cells showed distinct immunoreactivity. Staining was classified in: SCORE 1+ (staining that is faint/barely detectable), SCORE 2+ (staining that is weak/moderate), SCORE 3+ (staining that is intense/strong). Images were acquired with original magnification × 400.

### Human microarray dataset analysis

Data from the microarray datasets GDS3964 and GDS1375 were downloaded from the Gene Expression Omnibus (GEO, https://www.ncbi.nlm.nih.gov/geoprofiles) website. GDS3964 GEO profile dataset was obtained in xenotransplant melanoma metastasis models, comparing genes differentially expressed between tumor samples derived from a poorly metastatic parental cell lines, and from their highly metastatic derivatives. GDS1375 GEO profile dataset has been constructed based on the expression profile in normal skin samples (normal), benign skin nevi (benign nevi) and primary malignant melanoma (malignant). Statistical analysis was performed applying Mann-Whitney test. *p* < 0.05 was considered significant.

### Cell lines, transfection and viral infection

M14 and SKMEL24 human melanoma cell lines were purchased from American Type Culture Collection (ATCC, Manassas, VA). PLF2, SAN, M20, SKMel28, Sbcl1, A375, JR8, ME1007 human melanoma cell lines were established at the Regina Elena National Cancer Institute [22, 26, 27]. ME4405, ME4686, ME8959, and ME10538 human melanoma cell lines were established by Dr. Andrea Anichini (Istituto Nazionale Tumori, Milan, Italy), as previously described [22, 26, 27]. C32 and WM115 human melanoma cell lines were kindly donated by Dr. Meenhard Herlyn (Wistar Institute, Philadelphia, USA), and by Dr. Federica Di Nicolantonio (University of Turin, Turin, Italy), respectively [22, 26, 27]. ME1, ME70 and ME47 were provided by Dr. Andrea Anichini.

All cell lines were cultured in RPMI medium (Euroclone, Milan, IT) containing 10% inactivated fetal bovine serum (FBS) (Hyclone, Thermo Scientific, South Logan, UT), 2 mM L-glutamine (Euroclone), and antibiotics, as previously described [22, 26, 27]. Cell lines have routinely been tested for mycoplasma contamination.

Stable control (empty) and Bcl-2 (bcl-2) overexpressing clones were generated by M14 and A375SM-SC1 human melanoma cells as previously described [21, 25]. These cells were cultured in complete RPMI medium in presence of 1 mg/ml puromicine (Sigma–Aldrich, St Louis, MO).

1.5 × 10^5^ M14 cells were seeded and 24 h later transfected with a 50 nM pooled oligonucleotide mix against Sema5A (si-Sema5A) or scramble target (si-Ctrl) sequences (DharmaconRNA Technologies, siGENOMES MARTpool, Lafayette, CO, USA) using jetPRIME (Polyplus Transfection, Sébastien Brant Illkirch, FRANCE) following the manufacturer’s protocol. 48 h after transfection, Sema5A protein expression was assessed by Western blot analysis. For Sema5A protein expression, 1.5 × 10^5^ M14 cells were seeded and after 24 h were transfected with an empty vector (control plasmid, Ctrl) or Sema5A-Fc-His plasmid (2 μg), expressing the extracellular domain of the murine Sema5A protein (ECD-Sema5A), C-terminally fused to the Fc region of human IgG1 in addition to a 6X Histidine tag (a gift from Dr. Woj Wojtowicz Addgene plasmid #72161) [28] using jetPRIME (Polyplus Transfection) following the manufacturer’s protocol. Sema5A protein expression was assessed after 48 h of transfection by Western blot analysis. Stable Sema5A clones were obtained by transfecting M14 parental cell line with plasmid expressing the full length human Sema5A protein (Sema5A Protein Vector, pPM-C-HA, #PV057575, www.abmgood.com, Richmond, BC, Canada). The corresponding empty vector (#PV001) was used as control plasmid (empty). After transfection by JetPRime reagent (Polyplus transfection) according to the manufacturer’s protocol, M14 cells were cultured in the presence of 1200 μg/ml geneticin. A375 and BV173 cells were transduced with lentiviral vector for inducible c-Myb silencing [29], cultured in DMEM medium (Euroclone,) containing 10% inactivated fetal bovine serum (FBS) (Hyclone), 2 mM L-glutamine (Euroclone), and antibiotics, and exposed or not to doxycycline (doxy, 1 μg/ml for 72 h, RPI corp. Mount Prospect, Illinois, USA). M14 cells were transfected with mimic miR-204 (mirVana miRNA Mimic, hsa-miR-204-5p, ThermoFisher) or mimic miRNA negative control (mirVana miRNA Mimic, Negative Control #1, ThermoFisher) at final concentration of 10 nM. INTERFERin transfection reagent (Polyplus Transfection) was used according to the manufacturer’s instructions. Mature miRNA was assayed 72 h after transfection by stem-loop PCR [30] and samples were normalized using RNU19 as endogenous control. RNA quantification of mature miR-204 expression was performed by quantitative RT-PCR using TaqMan, Universal PCR Master Mix No AmpErase UNG (Applied Biosystems by Thermo Scientific).

### Western blotting analysis

Antibodies directed to Sema5A (#AP12324PU-N, OriGene, Rockville, Maryland, US), AKT (#9272S Cell Signaling, Danvers, MA, USA), phosphorylated AKT (#9271S, Cell Signaling), p44/42 (ERK1/2, #9102, Cell Signaling), phosphorylated p44/42 (ERK1/2, #9106 L, Cell Signaling), c-Myb (#AB45150, Abcam, Cambridge, MA, USA) were used. β-actin (#A1978, Sigma-Aldrich) and HSP70/72 (#HSP01, Calbiochem, San Diego, CA, USA) were used to check equivalent transfer and loading. Antibody binding was visualized by enhanced chemiluminescence method (Pierce ECL Plus Western Blotting Substrate, Thermo Scientific) according to manufacturer’s specification. The densitometric evaluation was performed using Image J software and normalized with relative controls.

### Cell migration and invasion assays

M14 cells, after 48 of transfection, were subjected to cell migration and invasion assays for 8 h as described [22]. Cell migration was also evaluated after pharmacological inhibition of MEK. 48 h after transfection, M14 cells were left untreated or treated with 10 nM Trametinib (GSK1120212, Selleckcem, Munich, Germany) for 6 h, then trypsinized and subjected to cell migration assays for 18 h.

### Vasculogenic mimicry (VM)

In vitro vasculogenic mimicry assay was performed seeding 1 × 10^5^ melanoma cells in serum-free medium onto the gelled basement matrix extracts, as described [22].

After 6 h, VM formation was photographed using light microscopy and quantified by counting the number of capillary-like structures in 10 set of images for each clone. Each clone was analyzed in duplicate in three different experiments. Statistical analysis was performed applying Student’s t-test. *p < 0.05* was considered significant.

### In vivo experiments

Control (empty) and Bcl-2 (bcl-2) overexpressing cells in exponential growth phase were harvested from the culture, washed, and resuspended in PBS and 5 × 10^6^ viable cells/mice were intramuscular injected into female CD-1 nude (nu/nu) mice (Charles River Laboratories, Calco, Italy) [31]. Mice were sacrificed 15 days after tumor injection and Sema5A expression in tumor sections was evaluated using Sema5A polyclonal antibody following the procedure described for the analysis of patient specimens. Representative images of immunohistochemical expression of Sema5A in empty and bcl-2 overexpressing xenografts were acquired by microscopy by original magnification × 400. All procedures involving animals and their care were authorized and certified by D.lgs 26/2014 (816/2015-PR of 11/08/2015) of the Italian Minister of Health.

### Quantitative real-time polymerase chain reaction (qRT-PCR) analysis

Total RNA was extracted from cultured cells using a Qiagen RNeasy Mini kit (Qiagen, Redwood City, CA, USA) according to the manifacturer’s instructions. Reverse transcription was performed using RevertAid Reverse Transcriptase (Thermo Scientific). qRT-PCR was performed with a Gene-Amp 7900 sequence detection system (Applied Biosystems, Foster City, CA, USA), using the SYBR green dye detection method. The mRNA levels were normalized using β-actin (*ACTB*) transcript. Relative mRNA levels were measured using the 2-Δ cycle threshold (2-ΔCT) method. Primers used to analyze each gene were: Sema5A: 5’-ACTGTTCTAGCGACGGCACC-3′ (forward), 5’-CCCCAGAAAGCCCATCTGT-3′(reverse), c-Myb: 5’-AAGTCTGGAAAGCGTCACTTG-3′ (forward), 5’-ACATCTGTTCGATTCGGGAGATA-3′ (reverse), β-actin: 5’-ATTGCCGACAGGATGCAGAA-3′ (forward), 5’-GCTGATCCACATCTGCTGGAA-3′ (reverse). Student’s t-test was used and results were considered significant if *p < 0.05.*

### mRNA decay measurement

Actinomycin D (10 μg/ml, Sigma-Aldrich) was added to control (empty) and Bcl-2 (bcl-2) overexpressing cells to inhibit mRNA transcription and to assess the stability of the Sema5A mRNA. Total RNA was extracted at 0, 90, 180, and 360 min after treatment. The relative amount of specific mRNA remaining in each sample at specified time points can be correlated with mRNA degradation [32]. The endogenous Sema5A mRNA levels were analysed by qRT-PCR. Since the mRNA level for *ACTB* did not change after Actinomycin D treatment, the *ACTB* gene was used as a reference gene, and the ratio of Sema5A and *ACTB* in each sample was calculated.

### Chromatin Immunoprecipitation (ChIP) Assay

Cross-linked chromatin was immunoprecipitated with anti-c-Myb (ChIP grade, #AB45150, Abcam) or anti-acetyl histone H3 PAN (#06–599, Millipore, Darmstadt, Germany) antibodies as described [33]. The genomic regions in the Sema5A and cyclin B1 (CCNB1) promoters were amplified using specific primers (Additional file 1: Table S1). Quantization of immunoprecipitated DNA was performed in triplicate using the SYBR green dye detection method.

### Statistical analysis

Values were presented as mean ± standard error of the mean (SEM) or mean ± standard deviation (SD), with a minimum of three replicates, unless specified. Results were evaluated by Student’s t-test, with *p < 0.05* considered significant.

## Results

### Sema5A affects in vitro melanoma cell invasion, migration and vasculogenic mimicry (VM)

**Fig. 1:**
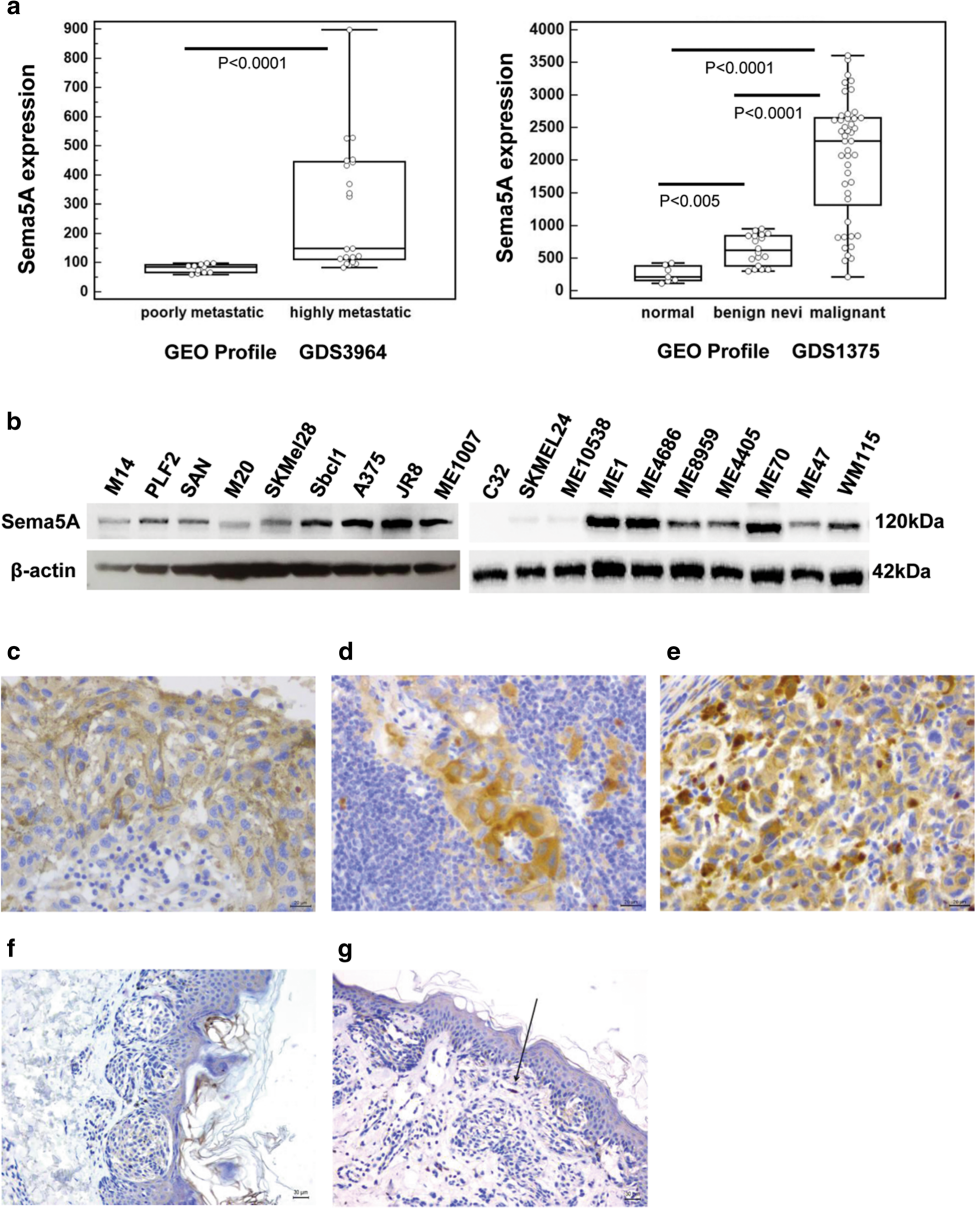
Evaluation of Sema5A expression in melanoma. **a** Box and whisker plots showing data from the microarray datasets GDS3964 and GDS1375 downloaded from the Gene Expression Omnibus (GEO, https://www.ncbi.nlm.nih.gov/geoprofiles) website (see Methods for details). Values of Sema5A gene expression levels are reported as counts. Statistical analysis was performed applying Mann-Whitney test. **b** Western blotting analysis of Sema5A protein expression in melanoma parental cell lines. Reported image is representative of two independent experiments with similar results. β-actin expression was evaluated as control of equivalent transfer and loading. **c**-**g** IHC analysis of Sema5A expression in human melanoma specimens. **c**-**e** Three representative samples showing different levels of Sema5A selected from metastatic specimens obtained from melanoma patients: **c** SCORE 1+ (staining that is faint/barely detectable), **d** SCORE 2+ (staining that is weak/moderate), **e** SCORE 3+ (staining that is intense/strong). Scale bar 20 μm. **f**, **g** Representative images from in situ melanoma specimens showing no immunoreactivity **f** or focal positivity **g** for Sema5A expression. Arrow indicates positive cell. Scale bar 30 μm

By interrogating Protein Atlas database (https://www.proteinatlas.org) we observed that Sema5A protein and mRNA are expressed in all tested cancer types, including melanoma. Since little is known regarding the role of Sema5A in melanoma, except for its detection in membrane preparations [9], we evaluated Sema5A expression in melanoma, by analysing the gene expression profiles. As shown in Fig. 1a, a significant up-regulation of Sema5A expression was observed in tumors derived from highly metastatic human cells, and increased Sema5A levels were observed in malignant samples compared to benign nevi. Moreover, as shown in Fig. 1b, Sema5A protein is expressed, although at different levels, in 18 out 19 (95%) human melanoma cell lines analysed. We also evaluated the expression of Sema5A protein by immunohistochemistry (IHC) in metastatic lesions from 13 patients with melanoma, finding 70% of positive specimens (Fig. 1c-e). In particular, 2 cases showed SCORE 1+, 4 cases showed a moderately staining (SCORE 2+), and 3 cases were highly positive (SCORE 3+). On the other hand, immunoreactivity was not observed in 10/12 (Fig. 1f) human in situ melanoma lesions, while two cases showed a focal positivity (SCORE 1+) in a < 1% of melanoma cells (Fig. 1g).

**Fig. 2:**
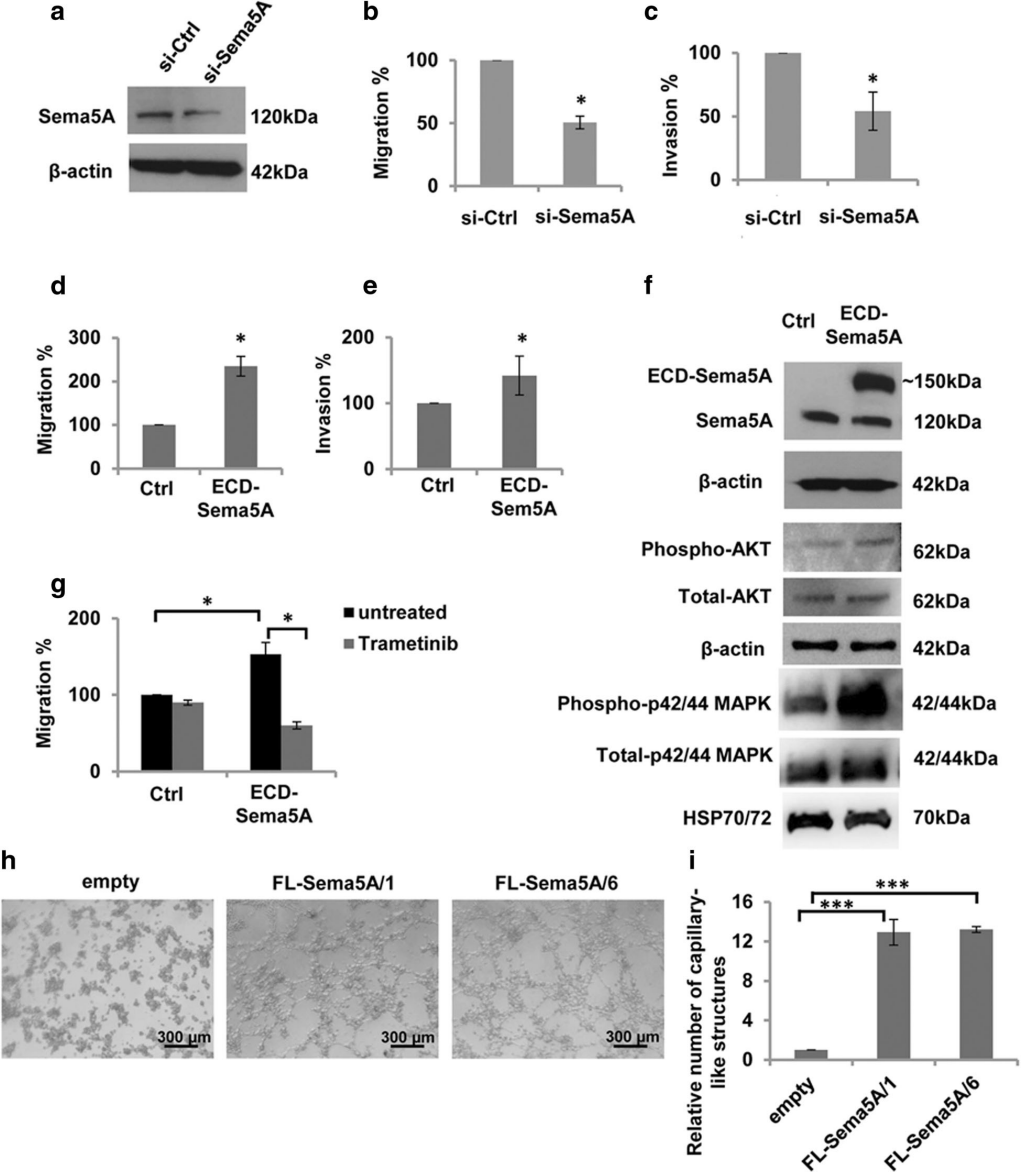
Sema5A affects in vitro melanoma cell invasion, migration and vasculogenic mimicry. **a** Western blotting analysis of Sema5A expression in M14 cells after 48 h transfection with pooled oligonucleotide mix against Sema5A (si-Sema5A) or scramble target (si-Ctrl) sequences. β-actin was evaluated as control of equivalent transfer and loading. Reported images are representative of three independent experiments with similar results. **b** In vitro cell migration and **c** invasion assay performed in M14 cells transfected with si-Ctrl or si-Sema5A. Values are presented as percentage of migrated/invaded cells in si-Sema5A versus control cells. **d** In vitro cell migration and (**e**) invasion assay performed in M14 cells transfected with empty vector (Ctrl) or vector expressing the Sema5A protein (ECD-Sema5A). Values are expressed as a percentage of migrated/invaded cells in ECD-Sema5A versus control cells. **b**-**e** Data were expressed as average ± standard deviation. *p-values < 0.05. **f** Western blotting analysis of Sema5A, total and phosphorylated AKT, total and phosphorylated p44/42 protein expression in M14 cells transfected with Ctrl and ECD-Sema5A vectors. Representative images of three independent experiments are reported. β-actin or HSP70/72 expression was evaluated as control of equivalent transfer and loading. **g** In vitro cell migration performed in M14 cells transfected with Ctrl or ECD-Sema5A, untreated or treated with 10 nM Trametinib. Values are expressed as a percentage of migrated cells in ECD-Sema5A versus control cells. Data are expressed as average ± standard deviation. *p-values < 0.05. **h** Representative images and **i** quantification of capillary-like structure formation in M14 melanoma clones overexpressing Sema5A protein (FL-Sema5A/1 and FL-Sema5A/6) and corresponding control clone (empty). Data were expressed as average ± standard deviation. ***p-values < 0.001. Scale bar, 300 μm

These results and the lack of published data on the relevance of Sema5A on melanoma prompted us to evaluate the role of Sema5A in melanoma aggressiveness. To this purpose, we performed in vitro transwell migration assays of Sema5A-silenced M14 cells. As depicted in Fig. 2, Sema5A downregulation (Fig. 2a) induced a decrease in migration (Fig. 2b, Additional file 2: Figure S1) and invasion (Fig. 2c, Additional file 2: Figure S1) of about 50%, when compared to cells transfected with control siRNA. We confirmed the role of Sema5A in regulating cell migration and invasion in M14 cells transiently transfected with a plasmid expressing the extracellular domain of Sema5A (ECD-Sema5A). As shown in Fig. 2, the number of migrating (Fig. 2d, Additional file 2: Figure S1) and invading (Fig. 2e, Additional file 2: Figure S1) cells was significantly higher in Sema5A-overexpressing cells, compared to control ones. These results indicate that Sema5A regulates aggressiveness-associated properties of melanoma cells.

To identify a signalling network mediating Sema5A migration and invasion, we examined the effect of Sema5A overexpression on phosphorylation of Akt and ERK1/2 (p42/44 MAPK) [34]. Fig. 2f shows that overexpressing Sema5A increased levels of Akt and p42/44 ERK1/2 phosphorylation. Treatment of ECD-Sema5A-transfected melanoma cells with Trametinib, a specific MEK inhibitor used for melanoma therapy [35, 36], significantly reduced the ability of Sema5A- overexpressing cells to migrate (Fig. 2g, Additional file 2: Figure S2), thus indicating that Sema5A-dependent cellular migration is mediated by activation of MEK pathway.

We further investigated the potential role of Sema5A on VM, a process that reflects the plasticity of aggressive melanoma cells by forming de novo vascular networks and is associated with the malignant phenotype and poor clinical outcome [37, 38]. To this end, we evaluated the formation of vascular channels after seeding melanoma cells (Additional file 2: Figure S3) onto the gelled basement matrix extracts. As shown in Fig. 2h and Fig. 2i, two Sema5A-overexpressing clones showed enhanced VM, evaluated on the number of capillary-like structures, when compared to the control clone. Enhanced VM was associated to increased secretion of VEGF protein, determined by ELISA, in two Sema5A transfectants, with a fold induction of 1.7 ± 0.2 and 1.8 ± 0.6 compared to controls.

### Bcl-2 modulates Sema5A expression in melanoma models

**Fig. 3:**
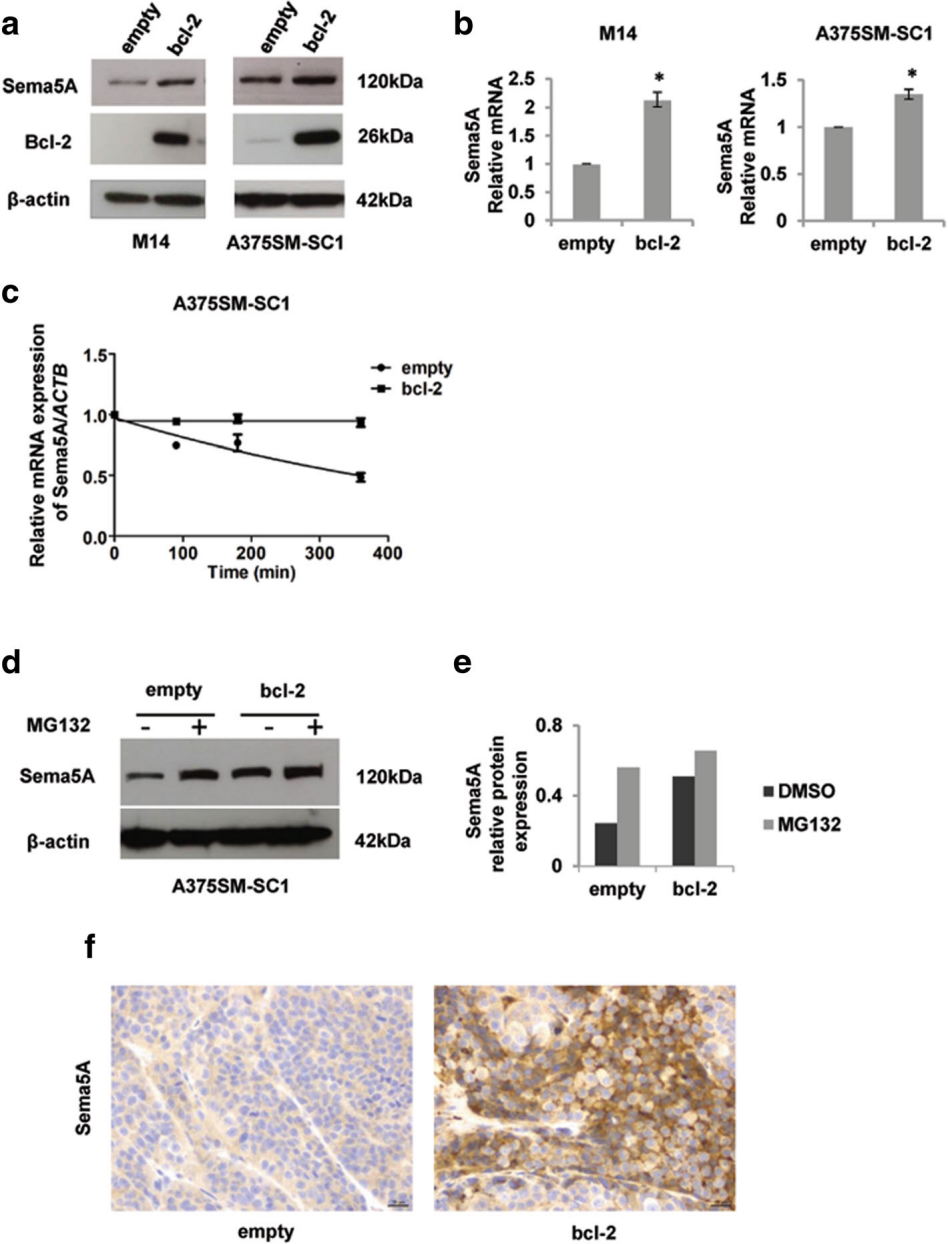
Bcl-2 modulates Sema5A expression in melanoma models. **a** Western blot analysis of Sema5A and Bcl-2 expression in M14 and A375SM-SC1 human melanoma control (empty) and Bcl-2 overexpressing (bcl-2) clones. β-actin was evaluated as control of equivalent transfer and loading. Reported images are representative of three independent experiments with similar results. **b** Analysis of Sema5A mRNA expression evaluated by qRT-PCR in the indicated cell lines. mRNA levels were normalized using β-actin. Values are expressed as means of ratio ± SEM where ratio was calculated considering Bcl-2-overexpressing versus control clones. Experiments were performed at least three times (three technical replicates). *p-values < 0.05. **c** Sema5A mRNA amount was evaluated by qRT-PCR in control (empty) and Bcl-2 overexpressing (bcl-2) clones after 0, 90, 180, and 360 min of treatment with Actinomycin D (10 μg/ml). The β-actin (*ACTB*) gene was used as a reference gene, and the ratio of Sema5A and *ACTB* in each sample was calculated. Data are shown as mean ± SD (*n* = 2). **d** Western blot analysis of Sema5A in A375SM-SC1 control (empty) and Bcl-2 overexpressing (bcl-2) clones treated or not with 10 μM MG132 (Sigma-Aldrich) for 6 h and **e** relative densitometric analysis performed using Image J software. Representative images of two experiments with similar results are reported. β-actin expression was evaluated to confirm equivalent transfer and loading. **f** Immunohistochemical analysis of Sema5A expression in M14 control (empty) and Bcl-2 overexpressing (bcl-2) xenografts. Representative images of Sema5A expression in empty (SCORE 1+) and bcl-2 overexpressing (SCORE 3+) xenografts are reported. Scale bar, 20 μm

By using M14 and A375SM-SC1 melanoma cell lines and their derivatives stably overexpressing *wild-type* Bcl-2 [25], we evaluated whether Bcl-2, a protein involved in melanoma progression, resistance to apoptosis, and poor prognosis [16], plays a role in the regulation of Sema5A expression. As shown in Fig. 3, increased Sema5A expression at both protein (Fig. 3a) and mRNA (Fig. 3b) level was found after Bcl-2 overexpression in both cell lines. By performing immunofluorescence and co-immunoprecipitation experiments, we excluded the possibility of cellular co-localization/interaction of Bcl-2 and Sema5A proteins (data not shown).

To investigate whether Bcl-2 has a role in regulating Sema5A transcription, we evaluated Sema5A transcripts at different times after treatment with Actinomycin D, a classical inhibitor of transcription elongation. As shown in Fig. 3c, exposure to Actinomycin D caused a significant reduction of Sema5A mRNA expression, while no appreciable decrease of Sema5A transcripts was detected in the Actinomycin D-treated Bcl-2-overexpressing cells. These results indicate the involvement of Bcl-2 in Sema5A mRNA stability.

We also analysed the level of Sema5A protein after treatment of A375SM-SC1 cells with MG132, a proteasome inhibitor. As shown in Fig. 3d,e, upon treatment with MG132, control cells showed an about two-fold increase of Sema5A protein level, while in Bcl-2 overexpressing cells the increase of Sema5A protein expression was more modest, indicating that Bcl-2 forced expression protects Sema5A protein from proteasome degradation. Most remarkably, the increment of Sema5A expression after Bcl-2 forced overexpression was also observed in in vivo experiments (Fig. 3f). Immunohistochemical analysis revealed higher Sema5A positivity in Bcl-2 overexpressing xenografts [31] compared to control xenografts.

### Sema5A is regulated by the miR-204/c-Myb axis

**Fig. 4:**
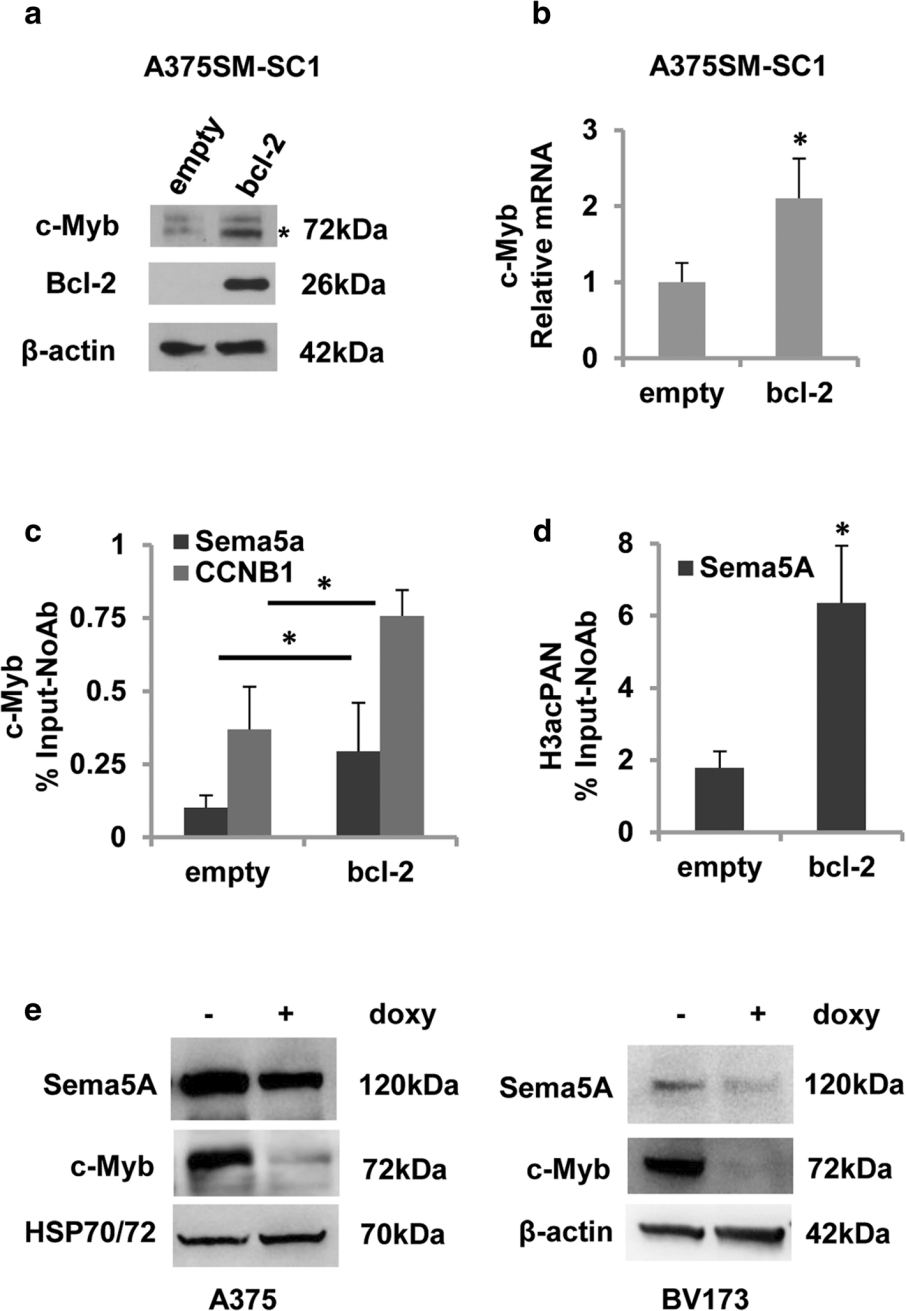
c-Myb binding to Sema5A promoter. **a** Western blot analysis of Bcl-2 and c-Myb expression and **b** qRT-PCR of c-Myb expression in A375SM-SC1 human melanoma control (empty) and Bcl-2 overexpressing (bcl-2) clones. mRNA levels were normalized using β-actin. Values are expressed as means of ratio ± SEM where ratio was calculated considering Bcl-2-overexpressing versus control clones. **c** c-Myb and **d** H3acPAN recruitment on Sema5A and Cyclin B1 (CCNB1) promoters by ChIP analysis. Values of each immunoprecipitated sample are expressed as percentage relative to their respective input and by subtracting the values obtained in the negative controls (no antibody).Values are expressed as ratio ± SEM where ratio was calculated considering Bcl-2-overexpressing versus control clones. *p-values < 0.05. **e** Western blot analysis of Sema5A and c-Myb expression in A375 and BV173 cells transduced with lentiviral vector for inducible c-Myb silencing. **a**, **e**, β-actin or HSP70/72 expression was evaluated to confirm equivalent transfer and loading. **b**, **c**, **d**, Experiments were performed at least three times (three technical replicates). *p-values < 0.05

To investigate the molecular mechanism responsible for Sema5A regulation, we interrogated bioinformatics tools (ALGGEN–PROMO, Gene Cards [Genomics for SEMA5A Gene, Transcription factor binding sites by QIAGEN in the SEMA5A gene promoter] and Lasagna) for prediction of putative transcription factor binding sequences in the Sema5A promoter. Superimposing results were obtained by three different sources, identifying a c-Myb binding site in the Sema5A promoter. As shown in Additional file 2: Figure S4, c-Myb protein is expressed in most of the analysed melanoma cell lines, and more importantly, Bcl-2 overexpressing A375SM-SC1 cells exhibit a higher level of c-Myb protein (Fig. 4a) and mRNA (Fig. 4b) when compared to control cells. Then, we investigated whether c-Myb binds directly to the Sema5A promoter. As control, we also amplified the promoter region containing the c-Myb binding site of cyclin B1 (CCNB1), a c-Myb target gene [39]. The results of ChIP analysis confirmed c-Myb recruitment to the Sema5A promoter (Fig. 4c). Interestingly, in agreement with western blots showing a higher level of c-Myb protein in Bcl-2 overexpressing cells (Fig. 4a), ChIP analysis showed that more c-Myb was bound to both Sema5A and CCNB1 promoters after Bcl-2 overexpression (Fig. 4c). To confirm the role of c-Myb in the transcription of Sema5A, we also evaluated the enrichment of acetylated histone H3 (H3acPAN), a marker of transcriptionally active chromatin, at the c-Myb binding site of the Sema5A promoter. ChIP analysis showed an enrichment of H3acPAN at the c-Myb binding site of the Sema5A promoter that was increased in Bcl-2 overexpressing cells, compared to controls (Fig. 4d). To further investigate the role of c-Myb in the regulation of Sema5A, we used A375 melanoma cells transduced with a lentiviral vector for doxycycline-inducible c-Myb silencing [29]. As shown in Fig. 4e, expression of Sema5A protein was downregulated after c-Myb silencing in A375 cells. Decreased expression of Sema5A was also observed in the BV173 human chronic myeloid leukemia (CML)-lymphoid blast crisis cells transduced with the same c-Myb lentiviral vector (Fig. 4e), confirming that c-Myb regulates the expression of Sema5A.

**Fig. 5:**
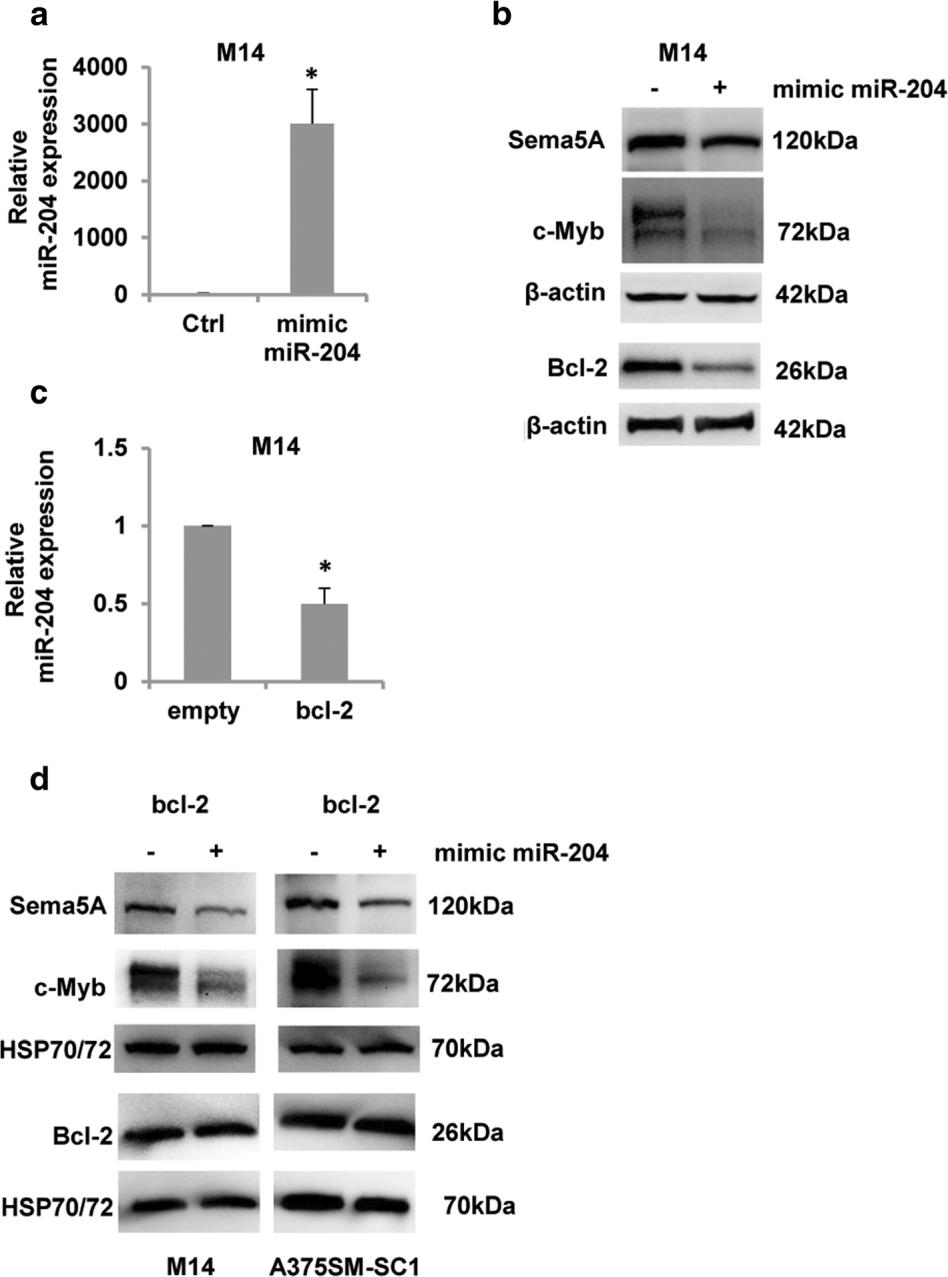
Sema5A is regulated by the miR-204/c-Myb axis. **a** Quantitative RT-PCR of miR-204 expression in M14 cells 72 h after transfection with mimic miR-204 or Pre-miRNA Precursor-Negative Control. Values are expressed as means of ratio ± SEM where ratio was calculated considering mimic miR-204 transfected cells versus control. **b** Western blot analysis of Sema5A, Bcl-2 and c-Myb expression after mir-204 transfection in M14 cells. **c** Quantitative RT-PCR of miR-204 expression in M14 human melanoma control (empty) and Bcl-2 overexpressing (bcl-2) clones. Samples were normalized using RNU19 as endogenous control. Values are expressed as means of ratio ± SEM where ratio was calculated considering Bcl-2-overexpressing versus control clones. *p-values < 0.05. **d** Western blot analysis of Sema5A, Bcl-2 and c-Myb expression after 48 h of miR-204 transfection in Bcl-2 overexpressing (bcl-2) clones from M14 and A375SM-SC1 cells. **b**, **d**, β-actin or HSP70/72 expression was evaluated to confirm equivalent transfer and loading. Representative images of three independent experiments are reported. **a**, **c** Experiments were performed at least three times (three technical replicates). *p-values< 0.05

A recent paper reported that miR-204, a microRNA involved in melanoma progression and response to chemotherapy [40, 41], regulates c-Myb expression in metastatic prostate cancer [42]. Thus, to investigate whether miR-204 regulates Sema5A expression through c-Myb, M14 cells were transiently transfected with mimic miR-204 (Fig. 5a) and the expression of both c-Myb and Sema5A was evaluated. Like in prostate cancer [42], c-Myb protein expression decreased after miR-204 overexpression, compared to control cells (Fig. 5b). Interestingly, a concomitant reduction of Sema5A protein expression was also observed in miR-204-transfected cells compared to the control counterpart (Fig. 5b), supporting the hypothesis that miR-204 plays an important role in regulating Sema5A expression. Overexpression of miR-204 also led to a decrease in Bcl-2 protein levels, in accordance with published data in gastric cancer [43].

We previously demonstrated that overexpression of Bcl-2 downregulates both miR-211 and miR-204 [25]. As displayed in Fig. 5c, M14 Bcl-2 overexpressing cells showed lower level of miR-204 respect to control cells and overexpression of synthetic miR-204 in both M14 and A375SM-SC-1 Bcl-2 transfectant is able to lower the level of both Sema5A and c-Myb proteins (Fig. 5d). These results indicate that the increased level of Sema5A and c-Myb proteins observed in Bcl-2 overexpressing clones can be due to the reduced level of miR-204 in these cells.

## Discussion

The aim of this study was to investigate the role of Sema5A [1, 44], in melanoma progression and to dissect the molecular mechanisms regulating its expression.

We focused our study on the poorly investigated Sema5A because, to the best of our knowledge, no data have been published yet on its expression and/or function in melanoma, but also because of its controversial role in cancer [9]. Indeed, Sema5A was found to reduce the motility of glioma cells [10], while having an opposite effect for the invasion of pancreatic, prostatic and gastric cancer cells [14, 45, 46].

In accordance with previously published studies in gastric, prostatic and pancreatic cancer, we show here that overexpression of ECD-Sema5A increased in vitro cell migration and invasion properties of melanoma cells, with a concomitant activation of the Akt/ERK pathways. By treating ECD-Sema5A-overexpressing cells with Trametinib, we demonstrated that Sema5A regulates cell migration through MEK/ERK activation. Moreover, Sema5A silencing confirmed the role of Sema5A in melanoma cell migration and invasion. The relevance of Sema5A in melanoma pathobiology is further supported by both public database of microarray profiling, showing higher level of Sema5A transcript in more aggressive forms of melanoma, and our findings that Sema5A protein is expressed in a panel of human melanoma cell lines and in metastatic melanoma specimens. On the contrary, “in situ” melanoma specimens showed only focal positivity in few cases. These results and data showing that Sema5A protein increases VM support the hypothesis that Sema5A expression is associated with melanoma aggressiveness.

As Bcl-2 protein plays a relevant role in melanoma, being associated with melanoma progression, resistance to apoptosis and poor prognosis [16, 17], we investigated the effect of Bcl-2 modulation on Sema5A expression. Bcl-2 forced expression in different melanoma models led to increased level of both Sema5A mRNA and protein. By use of transcription and proteasome inhibitors, we found that Bcl-2 is involved in the regulation of Sema5A transcript stability and protein degradation, respectively. In particular, Bcl-2 forced expression increased Sema5A RNA stability and made Sema5A protein less susceptible to proteasome-dependent degradation.

In agreement with findings in systemic lupus erythematosus patients that show a positive correlation between mRNA expression of ADAM17 and serum level of Sema5A [47], and with data in HeLa cells showing the cleavage of Sema5A by ADAM17 [48], we found that the increased amount of Sema5A levels after forced expression of Bcl-2, were paralleled by increased activation of ADAM17 (data not shown), thus suggesting that Bcl-2 might process Sema5A protein through ADAM17 activation.

We also provided evidence that Sema5A expression is under the control of the miR-204/c-Myb axis. c-Myb plays an essential role in regulating cell growth and differentiation of hematopoietic cells [49], promotes leukemic cell transformation [50], and is also involved in the development and progression of several solid tumors, including melanoma [51, 52]. More than 80 genes have been reported to be c-Myb targets, including Bcl-2 [29, 39]. Although at varying levels, c-Myb protein expression was detected in most human melanoma cell lines analysed. Interestingly, we found a higher level of c-Myb at both mRNA and protein levels in Bcl-2 overexpressing melanoma cells, and in accordance with this result, the recruitment of the c-Myb protein at the Sema5A promoter was enhanced by Bcl-2 forced expression. ChIP experiments also showed that, after Bcl-2 forced expression, more c-Myb was bound to the promoter of CCNB1, a well known c-Myb target gene [39]. The observed high acetylation level of the Sema5A promoter region containing the Myb binding site, and the decrement of Sema5A protein level observed after c-Myb knockdown in A375 melanoma cells and in BV173 CML-lymphoid blast crisis cells, confirmed the c-Myb dependent expression of Sema5A.

A regulatory loop of miR-204-5p and transcription factors, including c-Myb, relevant for malignant lineage development, was recently described [42]. miR-204-5p is down-regulated and acts as a tumor suppressor in different human tumor types, including melanoma [41, 53–55]. It also contributes to melanoma progression and resistance to BRAF, MEK and ERK inhibitors [41, 56]. On the basis of these findings, we evaluated the possible involvement of miR-204 in regulating Sema5A expression via c-Myb. By using a synthetic miR-204 we demonstrated that melanoma cells overexpressing miR-204 exhibit evident decreased levels of c-Myb together with downregulation of Sema5A, even if with less drastic affect, thus indication that, although we demonstrated for the first time the regulation of Sema5A by c-Myb and Bcl-2, other cellular factors may be involved in the regulation of Sema5A expression.

We also confirmed the ability of miR-204 to target Bcl-2, as previously reported in gastric cancer [43], thus corroborating the correlation between Bcl-2 and Sema5A expression, initially observed in Bcl-2 overexpressing clones. In accordance with these evidences we observed reduced level of miR-204 in Bcl-2 overexpressing cells, thus explaining the higher levels of both Sema5A and c-Myb protein found in these cells.

## Conclusion

**Fig. 6:**
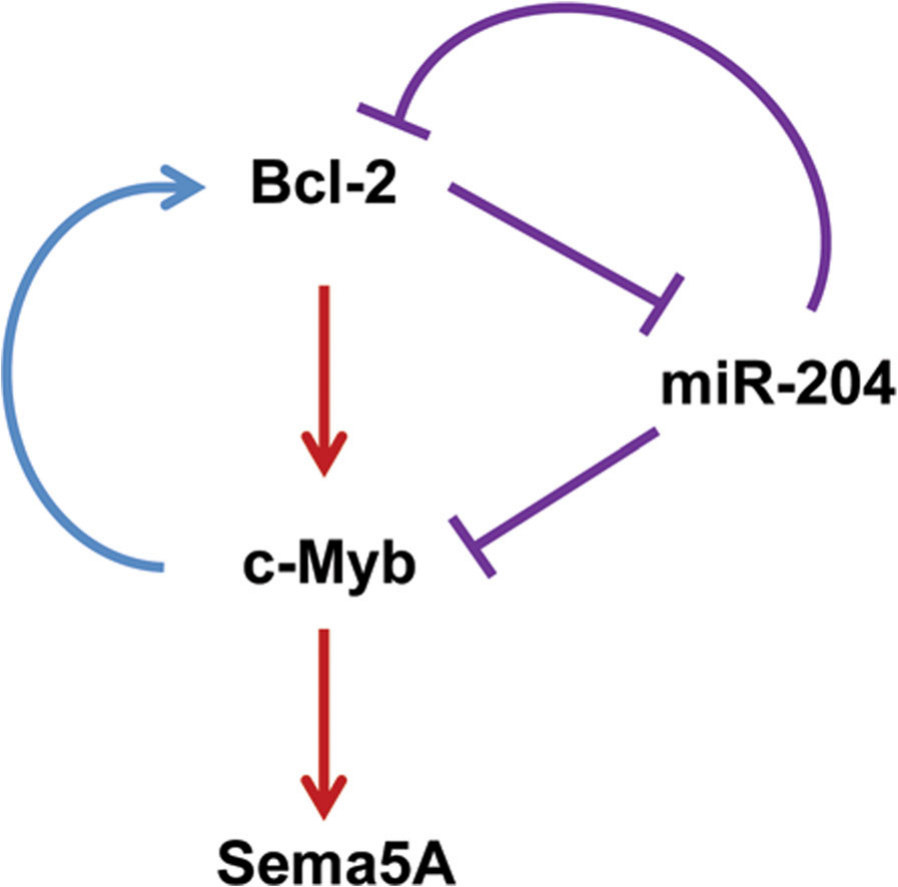
Schematic representation of the cellular network involved in the regulation of Sema5A expression. Blue line: evidences previously reported [29, 39]; violet lines: evidences previously reported [25, 42, 43] and confirmed in this work; red lines: evidences demonstrated in this work for the first time

Overall, our findings show that Sema5A promotes the in vitro migration and invasion of melanoma cells through Akt/ERK phosphorylation, increasing also VM. In addition, our data indicate that the miR-204/c-Myb axis is involved in the regulation of Sema5A expression and support the existence of a regulatory circuitry involving miR-204, c-Myb, and Bcl-2 (Fig. 6). Thus, Sema5A could represent a potential target for the treatment of melanoma.

## Additional files

Additional file 1: **Table S1.** Primer sequences used in ChIP analysis. (PDF 224 kb)

Additional file 2: **Figure S1.** Representative images of in vitro cell migration and invasion in scramble control (si-Ctrl) or Sema5A silenced (si-Sema5A) and plasmid control (Ctrl) or ECD-Sema5A overexpressing (ECD-Sema5A) M14 cells. **Figure S2.** Representative images of in vitro cell migration of plasmid control (Ctrl) or ECD-Sema5A overexpressing (ECD-Sema5A) M14 cells treated with 10 nM Trametinib or drug vehicle (untreated) for 6 h, prior performing migration assay. **Figure S3.** Western blotting analysis of Sema5A expression in M14 melanoma cells stably overexpressing the full-length Sema5A protein (FL-Sema5A/1 and Sema5A/6). HSP70/72 expression was evaluated to confirm equivalent transfer and loading. Representative images of two independent experiments are reported. **Figure S4.** Western blotting analysis of c-Myb protein expression in melanoma parental cell lines, cultured as previously reported [22, 26, 27]. Reported images are representative of two independent experiments with similar results. β-actin expression was evaluated to confirm equivalent transfer and loading. Representative images of two independent experiments are reported. (PDF 630 kb)

## Abbreviations

ChIP: Chromatin immunoprecipitation
doxy: Doxycycline
HSP70/72: 70/72 kDa heat shock protein
IHC: Immunohistochemistry
MMP-9: Matrix metalloproteinase-9
mRNA: Messenger RNA
qRT-PCR: Quantitative real-time PCR
S.E.M: Standard error of the mean
SD: Standard deviation
VEGF: Vascular Endothelial Growth Factor
