# Supplementary material for: Semaphorin 5A drives melanoma progression: role of Bcl-2, miR-204 and c-Myb
Source: J Exp Clin Cancer Res. 2018 Nov 19;37:278. doi: 10.1186/s13046-018-0933-x (PMC6245779; doi:10.1186/s13046-018-0933-x)
Supplement: Supplementary file 1 — Table S1. Primer sequences used in ChIP analysis. (PDF 224 kb) [file 13046_2018_933_MOESM1_ESM.pdf]

TABLE S1

Primer sequences used in ChIP analysis

Promoter (gene)	Foward	Reverse
**Sema5A**	5’-CAGCGCTCTCCGCCCCGCGCT-3’	5’-CGCTCGGGAGCGGGCTCAGG-3’
**CCNB1**	5’-TGTGAGGCCAAGAGTTTAAGA-3’	5’-ATCATAGCTCACTGTAACCTC-3’
