# Supplementary figures and images for: Semaphorin 5A drives melanoma progression: role of Bcl-2, miR-204 and c-Myb
Source: J Exp Clin Cancer Res. 2018 Nov 19;37:278. doi: 10.1186/s13046-018-0933-x (PMC6245779; doi:10.1186/s13046-018-0933-x)

Fig. S1

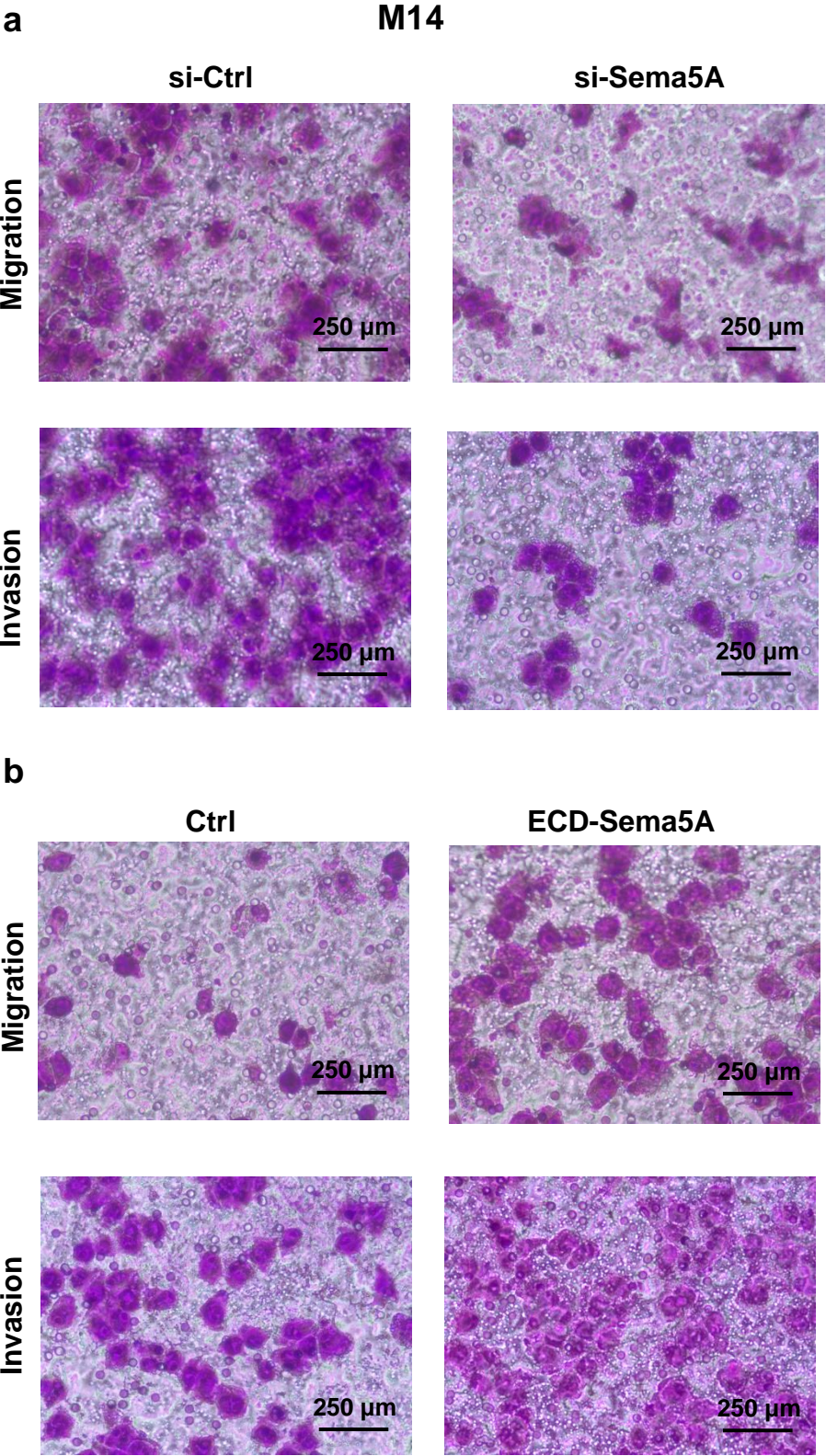

Fig. S2

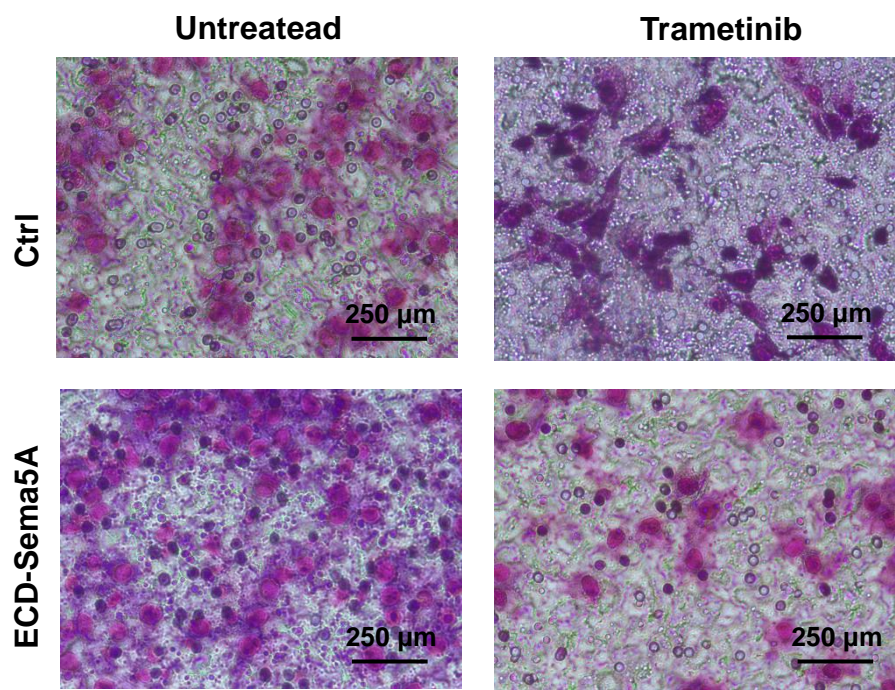

Fig.S3

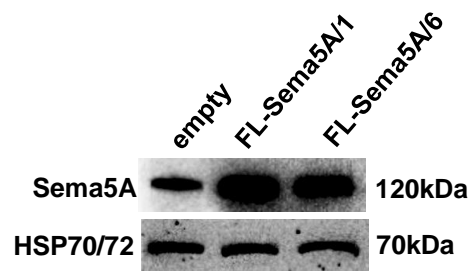

Fig. S4

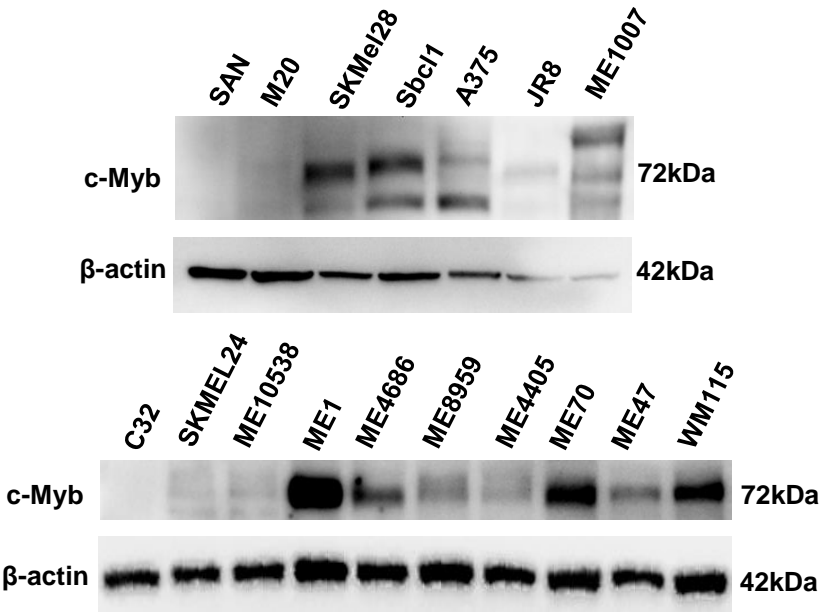

Supplement: Supplementary file 2 — Figure S1. Representative images of in vitro cell migration and invasion in scramble control (si-Ctrl) or Sema5A silenced (si-Sema5A) and plasmid control (Ctrl) or ECD-Sema5A overexpressing (ECD-Sema5A) M14 cells. Figure S2. Representative images of in vitro cell migration of plasmid control (Ctrl) or ECD-Sema5A overexpressing (ECD-Sema5A) M14 cells treated with 10 nM Trametinib or drug vehicle (untreated) for 6 h, prior performing migration assay. Figure S3. Western blotting analysis of Sema5A expression in M14 melanoma cells stably overexpressing the full-length Sema5A protein (FL-Sema5A/1 and Sema5A/6). HSP70/72 expression was evaluated to confirm equivalent transfer and loading. Representative images of two independent experiments are reported. Figure S4. Western blotting analysis of c-Myb protein expression in melanoma parental cell lines, cultured as previously reported [22, 26, 27]. Reported images are representative of two independent experiments with similar results. β-actin expression was evaluated to confirm equivalent transfer and loading. Representative images of two independent experiments are reported. (PDF 630 kb) [file 13046_2018_933_MOESM2_ESM.pdf]
